# Placental Microbial Colonization and Its Association With Pre-eclampsia

**DOI:** 10.3389/fcimb.2020.00413

**Published:** 2020-08-12

**Authors:** Kehinde S. Olaniyi, Jagidesa Moodley, Yesholata Mahabeer, Irene Mackraj

**Affiliations:** ^1^Nelson R. Mandela School of Medicine, School of Laboratory Medicine and Medical Sciences, University of KwaZulu-Natal, Durban, South Africa; ^2^Women's Health and HIV Research Group, Nelson R. Mandela School of Medicine, College of Health Sciences, University of KwaZulu-Natal, Durban, South Africa; ^3^Department of Medical Microbiology, National Health Laboratory Service, University of KwaZulu-Natal, Durban, South Africa; ^4^Department of Microbiology, Nelson R. Mandela School of Medicine, University of KwaZulu-Natal, Durban, South Africa

**Keywords:** metabolism, microbe, immune tolerance, placental microbiome, pre-eclampsia, pregnancy

## Abstract

The existence and role of the microbiome in regulating physiological and pathophysiological conditions including metabolism, energy homeostasis, immune tolerance, behavior, obesity, diabetes, and cardiovascular-related diseases is of immense interest. It is now clear that the human placenta is not sterile, but rather colonized with microbes. The placental and vaginal microbiomes are distinct however, the placental microbiome is comparable with the oral microbiome, with a limited variation when compared with the gut microbiome. Pre-eclampsia (PE), a pregnancy-specific hypertensive disorder, remains the leading cause of maternal-fetal morbidity and mortality. This is largely due to the lack of a clear etiology of PE and consequently, diagnostic strategies, and treatment are sub-optimal. The present review focuses on the current understanding of the placental microbiome and its implication in the etiology of PE. It provides a perspective on the alteration of placental microbiome as a possible therapeutic approach in the prevention and management of PE.

## Introduction

The microbiome refers to trillions of microbes that reside in different parts of the human body including the oral cavity, nasal cavity, gut, lungs, genitourinary tract, amniotic fluid, and the placenta (Chierico et al., 2014; Amarasekara et al., 2015; Zhang et al., 2015; Kumar and Chordia, 2017; Pelzer et al., 2017). The composition of microbes in an individual host depends on the genetic constitution, dietary intake, disease state, geographical location, and the dominant microbial species (Bull and Plummer, 2014; Chierico et al., 2014; Gilbert et al., 2015), and is genetically diverse as no two microbiota are the same (Kumar and Chordia, 2017). It is an essential component of immunity, capable of influencing metabolism and modulating drug interactions (Tancrede, 1992; Bull and Plummer, 2014; Kumar and Chordia, 2017; Wang et al., 2018). Therefore, the microbiome plays a crucial role in maintaining human health. In fact, dysbiosis (microbial imbalance) through diet, excessive use of antibiotics, obsession with cleanliness, cesarean deliveries etc. have been documented to contribute to some common diseases such as autism, diabetes, obesity, cancer, autoimmune diseases, and asthma (Constante et al., 2017; Postler and Ghosh, 2017).

The uterus and placenta were thought to be germ-free and sterile, maintained by cervical mucus (plug) to keep the baby safe from infection until recently that the existence of the placental microbiome is emerging, specifically in 2014 when the historical view of the sterile uterus and placenta was challenged by Aagaard et al. (2014) who reported detection of bacterial DNA sequences in the placenta samples of term and preterm pregnancies. Evidence exists that a distinct community of microbes colonize the placenta and include *Lactobacillus* sp., *Propionibacterium* sp., *Firmicutes, Tenericutes, Proteobacteria, Bacteroidetes*, and *Fusobacteria phyla, Enterobacter* sp., *Salmonella* sp., *Porphyromonas* sp., *Klebsiella pneumoniae, Bacillus cereus, Gardnerella* sp., *Variovorax* sp., *Clostridium* sp., *Prevotella* sp., *Listeria* sp., *Escherichia* sp., *Anoxybacillus* sp., among others (Amarasekara et al., 2015; Gomez-Arango et al., 2017), many of which are usually associated with periodontitis and chorioamnionitis (Amarasekara et al., 2015; Gomez-Arango et al., 2017). Histological analyses have also revealed that intracellular bacteria are present in the basal plate of a term placental biopsy (Stout et al., 2013), and that bacteria home and replicate within the placental explants from a term pregnancy (Cao and Mysorekar, 2014). Similarly, a low-abundance of microbiome has been identified from the placentae of a healthy pregnancy using culture-dependent and -independent methods. Likewise, previous studies have suggested oral mucosa as a possible source of the placental microbiome based on the similarities between microbial communities in the placental and oral niches (Aagaard et al., 2014). Additionally, evidence of phyla-specific similarities of microbes in the placenta, infant meconium and amniotic fluid, it has been proposed that microbiota from the placenta *in utero* is the source of the complement of developing fetus (Collado et al., 2016), and detectable bacteria in the umbilical cord blood could also originate from the placenta (Jiménez et al., 2005; Goeden et al., 2013; Cox et al., 2014; Sedlmayr et al., 2014; Gomez-Arango et al., 2017). Collectively, these studies corroborate the existence of microbial communities in the placenta and that the fetal environment is not sterile. However, the involvement of placental microbiome in pregnancy complications particularly pre-eclampsia (PE) is still under investigation.

Pre-eclampsia, the leading cause of maternal-fetal morbidity and mortality (Maebayashi et al., 2014; Rana et al., 2019), is a multi-systemic syndrome that affects 5–8% of pregnancies worldwide, resulting in over 70,000 maternal and 500,000 fetal deaths annually (Wanderer et al., 2013; Goel et al., 2015). It is a pregnancy-specific disorder that is usually characterized by new-onset hypertension and proteinuria. It should be noted that PE can also be diagnosed without the presence of proteinuria but with evidence of thrombocytopenia and elevated liver enzymes. The precise cause of PE remains unknown and as a result, there are no clear screening tools or preventative measures for early diagnosis of PE (Amarasekara et al., 2015; Rana et al., 2019). Although the pathogenesis of PE is well-documented and maternal and fetal/placental factors have been studied extensively (Wanderer et al., 2013; Gathiram and Moodley, 2016; Karumanchi, 2018; Pillay et al., 2019; Rana et al., 2019), with placenta recognized as the central causative agent.

Abnormal placentation has been associated with uteroplacental ischemia early in the first trimester and followed by maternal syndrome in the later second and third trimesters. It is characterized by altered anti-angiogenic and pro-angiogenic factors, shedding of syncytiotrophoblast microparticles, and nanovesicles into the maternal circulation. These changes collectively drive the hypertensive, multi-organ failure response observed in the maternal pre-eclamptic syndrome (Palei et al., 2013; Romero and Chaiworapongsa, 2013; Verma et al., 2018; Pillay et al., 2019). A number of theories have been proposed for the placental dysfunction observed and include oxidative stress, abnormal natural killer cells (NKs) at the maternal-fetal interface, and genetic and environmental factors. Although none of these theories have conclusive evidence, substantive evidence supports the idea that the diseased placenta triggers the release of soluble toxic factors in the maternal circulation and these result in inflammation, impaired endothelial function and maternal systemic disease (Wanderer et al., 2013; Verlohren et al., 2014; Erez et al., 2017; Karumanchi, 2018). It is therefore suggested that the presence of bacteria in the placenta may also alter anti-angiogenic factors, such as soluble fms-like tyrosine kinase 1 (sFlt-1) and pro-angiogenic factors, like placental growth factors (PIGF), and vascular endothelial growth factor (VEGF). This results in an antiangiogenic state causing impaired maternal endothelial function that leads to the clinical manifestation of PE (Govender et al., 2012; Baijnath et al., 2014; Pillay et al., 2017). Because the exact cause of PE is unknown, its treatment has been limited to reduction of high blood pressure and delivery of placenta and fetus. The present review is focused on the current understanding of the placental microbiome and its implication in the etiology of PE and provides a perspective that placental microbiome-targeted therapy may be beneficial in the treatment of PE.

## The Sterile *In Utero*-Placental Environmental Paradigm Shift

The notion that the human *in utero*-environmental placenta is sterile, and that neonates acquire microbiome during and after birth was accepted dogma for more than a century (Escherich, 1885; Funkhouser and Bordenstein, 2013). Even to date, there are still controversies regarding *in utero/*placental microbial colonization. A recent study by Theis et al. (2019) and de Goffau et al. (2019) revealed that the human placenta has no potential microbes and these authors attributed the limited bacterial presence in the placenta to the contamination of used laboratory reagents with bacterial DNA or acquisition during labor and delivery. This view was also supported by successful generations of animals that are germ-free through aseptic transfer of the entire uterus reinforcing evidence against the existence of the placental microbiome (Hedrich and Hardy, 2012). However, increasing evidence in the last decade has revealed that the fetal environment is not sterile, and proof of the existence of *in utero/*placental microbial colonization is being reported (Jiménez et al., 2005; Aagaard et al., 2014; Parnell et al., 2017; Perez-Muñoz et al., 2017). Interestingly, using molecular techniques, recent studies confirmed that the bacterial communities which exist in the placenta and amniotic fluid from term pregnancies may vary from preterm pregnancies (Collado et al., 2016; Gomez-de Aguero et al., 2016). Studies also reveal *lactobacilli* and fastidious bacteria in the chorioamniotic membrane of term pregnancies, and that not all microbial colonization was linked with placental inflammation, chorioamnionitis, and infection (Lannon et al., 2019). This is consistent with earlier reports that the placenta of a healthy term pregnancy consists of non-pathogenic commensal bacteria (Aagaard et al., 2014). Importantly, others not only support the presence of placental microbiome but also found variance in microbiota with distinct genomic profiles based on the region of placenta they were obtained (Kumar and Chordia, 2017; Parnell et al., 2017). Collectively, these studies support a shift from a sterile uterus paradigm to *in utero* microbial colonization.

Nevertheless, Theis et al. recently reported an inconsistent results in the existence of microbial communities in the placental and fetal tissues using culture, qPCR, and 16S rRNA gene sequencing to profile the possible bacterial communities in the placental and fetal tissue of mice compared with maternal mouth, lung, liver, uterus, cervix, and vagina. Although the authors found a single bacterial isolate in the fetal brain sample having a bacterial load higher than that of contamination controls (Theis et al., 2020). These reports contradicted several early results in mice, including reports by Martinez et al., who found a diverse profile of bacteria in the placenta and concluded that fetuses are exposed to bacterial DNA *in utero* (Martinez et al., 2018), and Younge et al. who also reported the presence of *Lactobacillus, Escherichia, Enterococcus, Bacteroides*, and *Bacillus* in the placental and fetal tissues of the mice and confirmed fetal exposure to microbial communities from the placental and the extraplacental membranes *in utero* (Younge et al., 2019). Although Leiby et al. (2018) and de Goffau et al. (2019), was in agreement with other studies, but despite the use of multiple modes of microbiological inquiry, its observation might possibly be limited by the absence of multiple fluorescent *in situ* hybridization (FISH) to visualize the potential bacterial communities in the placental and fetal tissues of mice, and also by the absence of effective control such as the use of germ-free and wild-type mice. It is possible that the low biomass of bacterial communities in the placenta affects its detection by the existing technologies. Therefore, more studies using DNA-based techniques and microscopy with appropriate controls are required, but this should not dispel the understanding of the potential health benefits and diseases associated with placental microbiome.

## The Placental Microbiome—The Endogenous Microbial Community

Although findings from earlier cultivation-dependent studies significantly underestimated the existence of microbes in the placenta of healthy term and complicated pregnancies, it was likely due to difficulty to culture bacteria because of their preference for anaerobic environments (DiGiulio et al., 2010). However, using newer techniques such as Illumina sequencing, a comprehensive characterization of the placental microbiome in over 300 healthy, term, and preterm pregnancies was possible (Aagaard et al., 2014). This study detected a lowly abundant but “metabolically enriched” microbiome in 0.002 mg of bacterial DNA per 1 g of placental tissue isolated and it included *Cutibacterium acne, Neisseria lactamica, Fusobacterium* sp., *Rhodococcus erythropolis, Prevotella tannerae, Escherichia* sp., *Neisseria polysaccharea, Streptomyces avermitilis, Bacteroides* sp., and *Escherichia coli* in both normal healthy and complicated pregnancies (Aagaard et al., 2014).

Besides, a recent study found a high abundance of *Enterobacteriaceae* sp., *Cutibacterium acne* sp., and *Lactobacillus* sp. in the communities of placental microbes of healthy term deliveries (Gomez-de Aguero et al., 2016). In contrast, *Lactobacilli* sp. are less than other microbes in the placental tissues of preterm deliveries, supporting the role of *Lactobacilli* in positive pregnancy outcomes. Various studies have confirmed bacterial colonization in preterm pregnancies associated with chorioamnionitis and intrauterine inflammation (Aagaard et al., 2014; Romero et al., 2014; Prince et al., 2015). In one study, 80% of preterm births that occurred <30 weeks of gestation, had evidence of bacterial invasion (Mor and Kwon, 2015). Growing literature suggests that microbes possibly induce pregnancy complications, such as preterm birth through innate immune responses against the bacteria, causing excessive inflammation, and/or apoptosis at the maternal-fetal interface (Romero et al., 2014; Mor and Kwon, 2015). Likewise, *in vivo* models of experimental animals have shown that loading of bacteria or bacterial products in animals triggers preterm delivery (Romero et al., 2014), and currently the notion that bacterial colonization is a normal component of the uterus in pregnancy and non- pregnancy is being accepted (Mor and Kwon, 2015).

Moreover, the characterization of placental microbiome is currently under investigation. However, available data reveals that placental microbiome in normal pregnancy is characterized by both gram-positive and gram-negative bacteria with *Lactobacillus* dominance, which has largely been reported to confer a protective benefit (Gomez-de Aguero et al., 2016; Moreno et al., 2016). Whereas, alterations or unnatural shift (imbalance) in the composition of placental microbiota, known as dysbiosis, a condition that is highly dominated with *Bacteroides* and less of *Lactobacillus* (Bardos et al., 2019). This imbalance in placental microbiome, which can be triggered by diet, extrinsic stressors, including environmental stressors, antibiotic exposure, sleep disturbance, physical activity, and psychological stress and genetics has been reported to alter host immune response, leading to microbial-driven inflammation that underlie pregnancy complications, including preterm birth and PE among others (Gomez-de Aguero et al., 2016; Bardos et al., 2019).

## Seeding of Placental Microbiome

**Figure 1:**
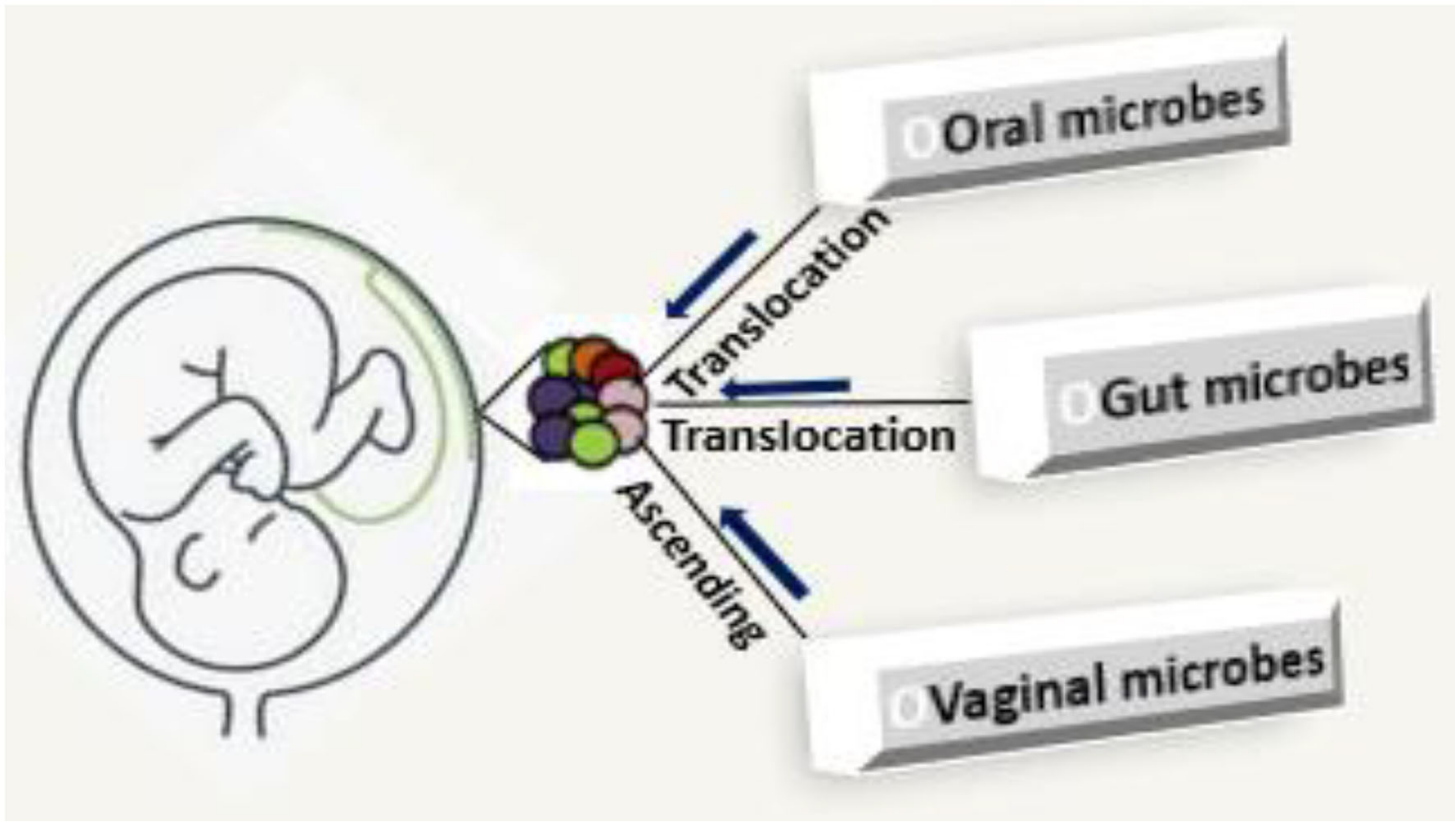
Seeding of placental microbiome; microbes ascend from the vagina, gut, and oral cavity which are internalized and translocated by hematogenous spread to the placenta.

The process of seeding of placenta microbiome in the current literature is not entirely clear. The present evidence is that: (i) microbes ascend from the vagina, and (ii) maternal intestinal lumen and oral cavity which are internalized and translocated by hematogenous spread to the placenta (Figure 1) or enter via maternal circulation to take up residence during early vascularization and placentation (Doyle et al., 2014; Antony et al., 2015; Gomez-de Aguero et al., 2016; Pelzer et al., 2017). The evidence for vaginal-derived bacteria is strong and *Lactobacilli* sp. from the vagina, are positively correlated with gestational age (Romero et al., 2015; Zheng et al., 2015; Pelzer et al., 2017). Translocation of bacteria from the epithelial gaps of the intestine and oral mucosa through the maternal circulation is enhanced during pregnancy and lactation, and this enables the transfer of low numbers of bacteria to possibly seed the placenta (Racicot et al., 2013; Prince et al., 2015; Pelzer et al., 2017; Lannon et al., 2019). Oral microbiota is thought to be a key source of the placental microbiome (Gomez-de Aguero et al., 2016). Bacterial DNA detection from oral microbiota has been associated with preterm rupture of membranes, preterm birth, miscarriage, and death of developing fetus for the genera pathogenic *Streptococcus* and *Fusobacterium* (Amarasekara et al., 2015), suggesting that oral microbiota is a contributor to the seeding of placental microbiome.

## Determination of Placental Microbiome

During the pre-sequencing era, culture was the most popular method used to determine the presence of microbes in the body, including placental microbes. This method however fails to detect viable but non-culturable microbes, supporting the sterile uterus paradigm. In contrast, the use of DNA-based techniques has upheld the existence of placental microbiome (Aagaard et al., 2014; Gomez-Arango et al., 2017). In addition, the use of real time PCR has successfully been used to profile placental microbiome. However, it has a low detection limit, therefore some viable bacteria may be undetectable. Community-wide composition profiling has been made possible by developmental approaches targeting the 16S ribosomal RNA (rRNA) molecule and its encoding gene. This is extremely useful to characterize the placental microbiota composition and its dynamics. In the last few years, 16S rRNA gene sequencing has become a popular approach to investigate the composition of the placental microbiota. Even more can be revealed at the DNA level, especially by the metagenomics approach in which a maximally representative DNA sample from the entire community of an environmental sample is isolated and subsequently, often in a random fashion, sequenced. Both the targeted 16S rRNA gene sequencing and shotgun metagenomic sequencing have been made possible with the onset of the next generation sequencing (NGS) techniques (Said et al., 2014; Yoneda et al., 2016; Urushiyama et al., 2017). Multiplexing is achieved via two strategies: adding a barcode sequence and through separate indexing reads, which are located outside the primer region, requiring separate sequencing runs (that read “away” from the target DNA molecule). For throughput, libraries are prepared for each sample with universal primers targeting different regions of the 16S rRNA gene. Primers are adapted for high-throughput sequencing with the addition of adapter sequences and barcoded dual-index forward and reverse sequences taken from previous studies (Aagaard et al., 2014; Zheng et al., 2015, 2017; Parnell et al., 2017). The processing of the data generated by NGS depends on the available bioinformatics capacities. Therefore, the DNA-based technique has greatly expanded our current knowledge of the placental microbiome. Other methods are still required to better understand the actual function and activity of microbes (metatranscriptomics, metaproteomics, and metabolomics) in the placenta.

## Significance of Placental Microbiome

**Figure 2:**
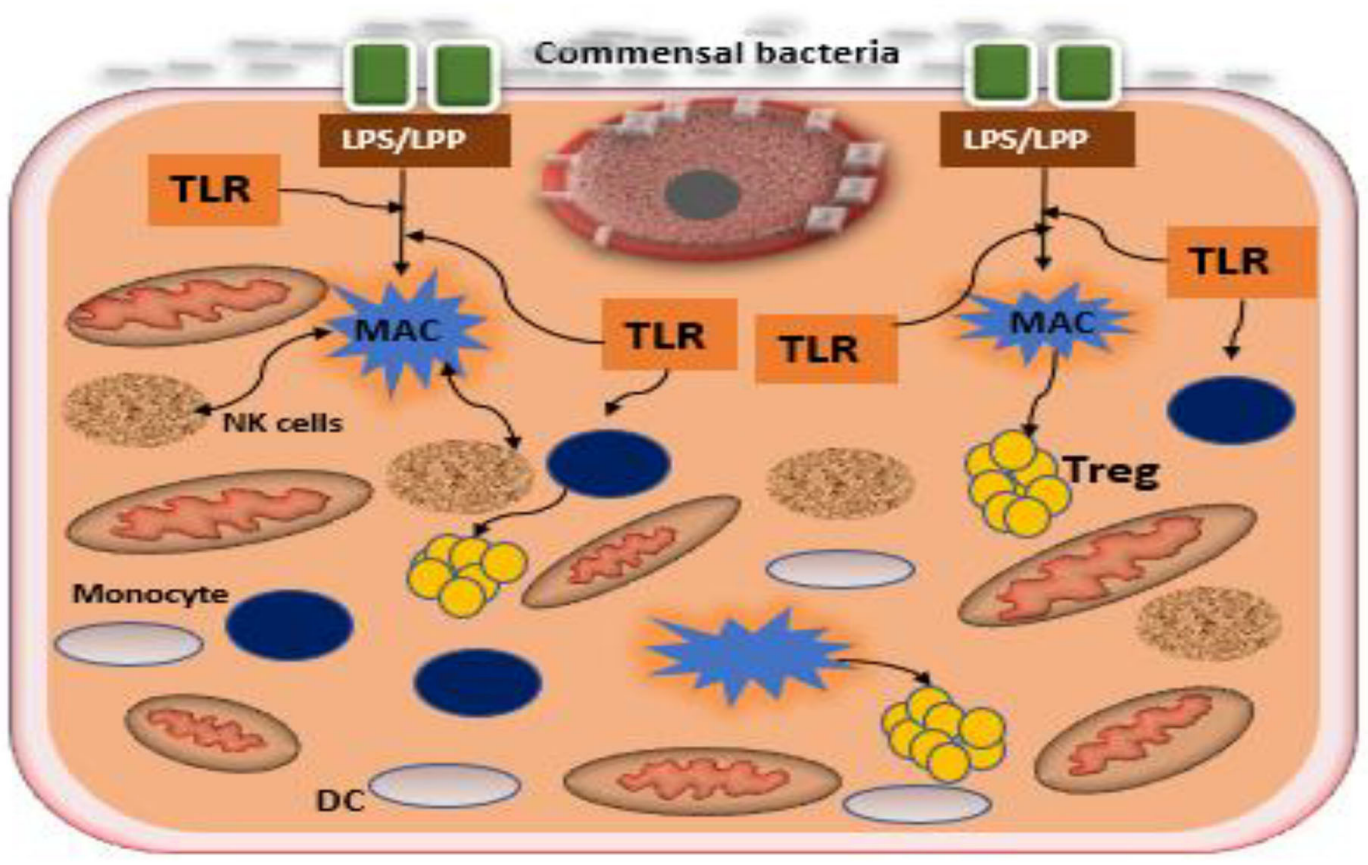
Interaction between commensal bacteria and toll like receptors of the trophoblast in the formation of regulatory cytokines. Commensal bacteria present at the epithelium of the uterus promote the induction of regulatory cytokines by trophoblast and macrophages. Macrophages secrete antimicrobial products that mitigate commensal overgrowth and prevent invasion of pathogenic bacteria. Recognition of bacterial products such as LPS or LPP by trophoblast potentiates the expression of anti-inflammatory factors, increasing T regulatory cells (Tregs), and promoting tolerance. TLR, toll-like receptor; DC, decidual cell; LPS, lipopolysaccharide; MAC, lipoprotein macrophage.

Several reports support the fact that the placental microbiome, particularly with non-pathogenic commensal dominance, is part of normal healthy pregnancy (Romero and Chaiworapongsa, 2013; Aagaard et al., 2014; Mor and Kwon, 2015; Zheng et al., 2017). *In utero* or placental microbial colonization is postulated to be involved in the following functions:

- Maternal immune modulation and initiation of early innate immune development in the fetus. The bacterial genetic diversity that is present in the host has generated important information regarding the immune-modulatory potential of the placental microbiota. A number of placental microbial genes particularly non-pathogenic commensals has been suggested to positively correlate with maternal-fetal immune tolerance (Racicot et al., 2013; Mor and Kwon, 2015). Microbiota modulate the host immune response by preventing an undesired inflammatory response during pregnancy thereby maintaining tissue homeostasis and maternal-fetal immune tolerance (Belkaid and Hand, 2014; Mor and Kwon, 2015; Gomez-de Aguero et al., 2016). Stimulation of toll-like receptors (TLRs) particularly TLR4 or TLR2 expressed on the trophoblast, by commensal bacteria, has been reported to suppress the NF-kB pathway (a classical inflammatory mediator), promoting the production of regulatory cytokines, such as type 1 interferon-associated chemokines and interleukin 10 (Figure 2). It is therefore plausible that bacteria might stimulate the maternal-fetal interface to a tolerogenic microenvironment through the induction of regulatory cytokines by the trophoblast (Mor and Kwon, 2015). Although the integration of microbial-derived signals by trophoblast remains unclear, epigenetic, and histone modifications or alternative pathways mediating response to TLRs have been recently suggested (Pelzer et al., 2017). In addition, the placental microbiota might be involved in driving early innate immune development and is emerging as a source of antigenic determinants in the newborn (Pelzer et al., 2017; Giessen et al., 2020).
- Modulation of metabolic function. The existing bacterial communities in the placenta seems to be metabolically enriched with genes associated with fatty acid (particularly short-chain fatty acid), tryptophan, and benzoate metabolism. Placental tryptophan metabolism is essential for fetal neural development and impaired placental tryptophan metabolism has been associated with neurodevelopmental defects in the fetus (Gomez-de Aguero et al., 2016). In effect, catabolism of tryptophan in the placenta enhances the establishment and maintenance of maternal-fetal immune tolerance (Sedlmayr et al., 2014), placental circulation, growth, and modulation of antimicrobial activity against infections (Sedlmayr et al., 2014). Placental bacteria are also enriched with pathways encoding fatty acid metabolism, which may aid energy extraction from circulating fatty acids and play an important role in supplying energy-yielding substrates to the fetus. Likewise, bacterial genes attributable to pathways involved in benzoate metabolism were also enriched in the placenta and aromatic compounds, for instance, benzoates are used as a carbon source for many microorganisms (Gomez-de Aguero et al., 2016).
- Preparation of the newborn for host-microbial symbiosis. Interaction of the microbes with human host is a significant contributor to pregnancy outcome and seeding of microbial colonization and molecular transfer in the neonates. Animal studies also support a role for neonatal immune priming and development without environmental microbial exposure (Gomez-de Aguero et al., 2016).

## The Possible Involvement of Placental Microbiome in Pre-Eclampsia

In order to understand the possible involvement of placental microbiome in PE, it is important to reiterate its spectrum and pathogenesis. Pre-eclampsia is one of the categories of the hypertensive disorder of pregnancy, which includes gestational hypertension, chronic hypertension, PE without severe features, severe PE, eclampsia, and the HELLP syndrome (USAID/Africa Bureau, 2012; Tranquilli et al., 2014). Besides, PE is clinically subtyped based on gestational age into early- and late-onset PE (Tranquilli et al., 2013; Magee et al., 2014; Mayrink et al., 2018). Early-onset PE is defined by new-onset hypertension before or at 33 weeks plus 6 days of gestation while late-onset PE is defined by new-onset hypertension at or after 34 weeks of gestation (Poon et al., 2010; Magee et al., 2014; Mayrink et al., 2019). Severe clinical manifestations of PE results in increased admission to intensive care units and may warrant early fetal-placental delivery to prevent complications that might result in fetal or maternal death (Jeyabalan, 2013; Magee et al., 2014).

To date the precise cause of PE is elusive and there is no screening test that has a high specificity and sensitivity to predict PE (Mayrink et al., 2019). However, several studies including earlier studies from our group have associated the pathogenesis of PE to maternal-fetal/placental factors (Ramesar et al., 2012; Gathiram and Moodley, 2016; Pillay et al., 2017; Karumanchi, 2018). The placenta seems to be a central agent in the etiology of PE and adequate research in this direction is essential. Nevertheless, the pathophysiological processes underlying PE have been primarily described in two stages. The first involves defective placental perfusion, possibly due to impaired placentation with abnormal trophoblast invasion and inadequate remodeling of the uterine spiral arteries, with placental hypoperfusion, hypoxia, and ischaemia resulting in decidual pathology. The second stage, referred to as the maternal syndrome, is characterized by systemic manifestations of inflammatory, metabolic, and thrombotic responses converging to promote vascular dysfunction which may lead to multi-organ damage (Ramesar et al., 2012; Baijnath et al., 2014; Mayrink et al., 2019). Cumulative evidence identified clinical risk factors (blood pressure, proteinuria, uterine-artery Doppler velocimetry, low platelet count, hemolysis, and elevated liver enzymes) and biochemical markers (pro-angiogenic and anti-angiogenic factors, cell free fetal DNA, cytokines, high-temperature-requirement A^3^ enzyme, placental proteins, elevated lipid profile among others) as predictive markers of PE (Gathiram and Moodley, 2016; Pillay et al., 2017, 2019; Karumanchi, 2018; Mayrink et al., 2019). Current data has recognized that placental-derived exosomes are potential biomarkers of PE (Pillay et al., 2017, 2019). Nevertheless, the etiology and an accurate screening test for early diagnosis, treatment and prevention, particularly in the nulliparous group that is at high risk of PE, remains elusive.

Immunological and metabolic maladaptations are the critical pathophysiological conditions associated with PE (Mor et al., 2011, 2017; Perez-Sepulveda et al., 2014; Bounds et al., 2015). Several studies have reported that early-onset PE is predominantly associated with an abnormal immune response (Laresgoiti-Servitje, 2013; Bounds et al., 2015; Mor et al., 2017; Lv et al., 2019), while late-onset is largely associated with metabolic perturbation owing to an imbalance between the metabolic demands of the developing fetus and maternal supply (Racicot et al., 2014; Verlohren et al., 2014; Erez et al., 2017; Lokki et al., 2018; Lv et al., 2019). However, considering the possible role of placental microbiome in the modulation of immune response and metabolic function in normal pregnancy (Belkaid and Hand, 2014; Mor and Kwon, 2015; Parnell et al., 2017; Pelzer et al., 2017; Giessen et al., 2020), we postulate that alterations in placental microbiome status contribute to PE through immunological and metabolic disruption. However, a growing body of data corroborates the concept of both uterine and placental communities of low biomass as discussed in the earlier part of the present review, but to predict and understand the contribution of the placental microbiome to PE, knowing the bacteria that are present matters less than knowing how the bacteria are capable of interacting with the host (Perez-Sepulveda et al., 2014; Mor et al., 2017). Interestingly, a recent analysis of placental gene expression in PE also implicates alteration in the expression of receptors on myeloid cells-1, metalloprotease INHA and lactotransferrin which correlate with changes obtainable during infection (Vigliani and Bakardjiev, 2014; Brew et al., 2016; Di Simone et al., 2017; Fillerova et al., 2017). These form the basis for the recent suggestion of antibiotics as a potential preventative measure for PE (Fillerova et al., 2017; Kenny and Kell, 2018).

## Role of Placental Microbiome in Tolerance and Placental Adaptation

**Figure 3:**
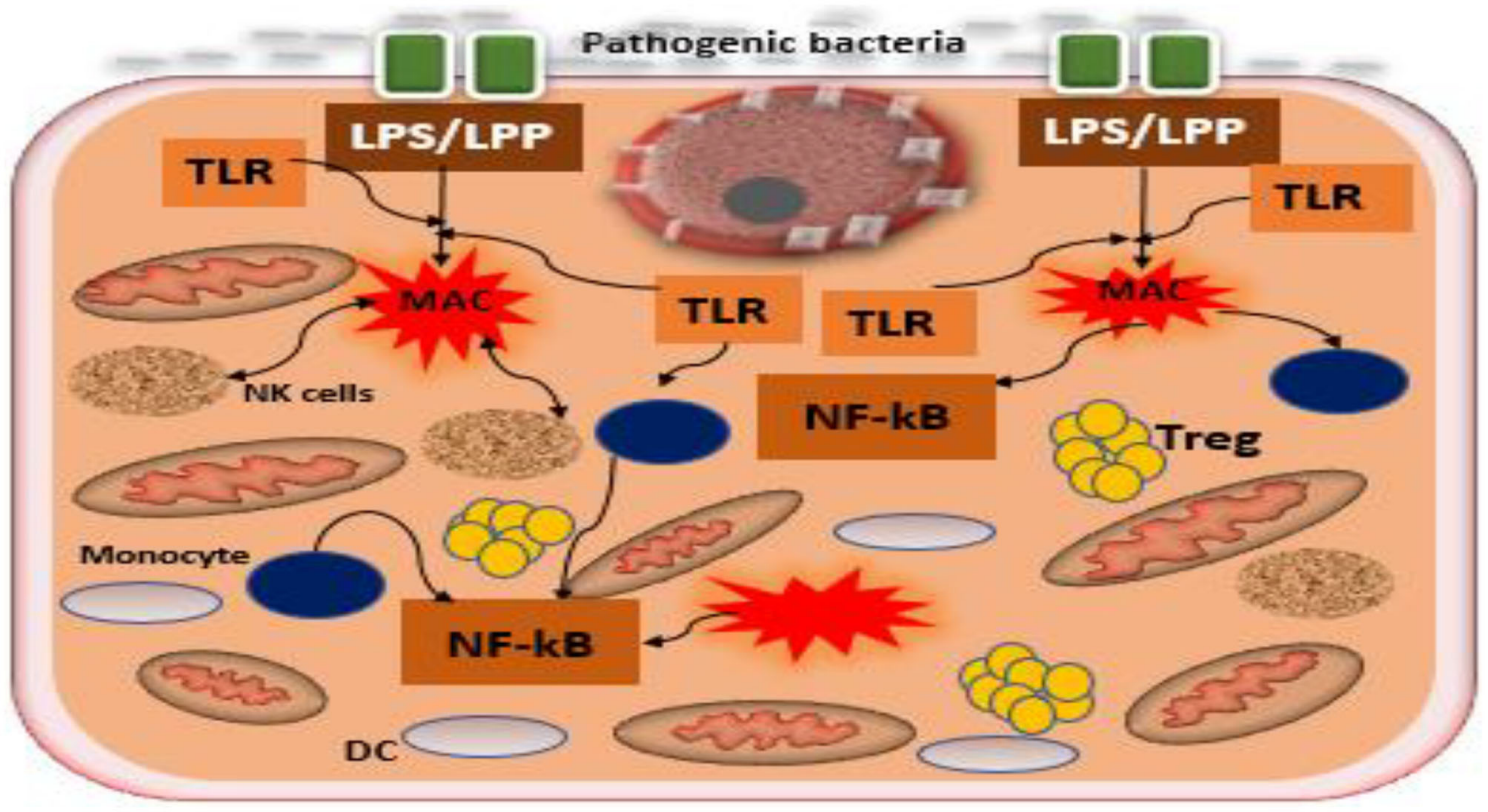
Interaction between pathogenic bacteria and toll-like receptors of the trophoblast in the formation of inflammatory cascades. Pathogenic bacteria, including the Gram-positive and Gram-negative present at the epithelium of the uterus promote the induction of inflammatory cascades by the interactions between the bacterial products LPP/LPS and toll-like receptors, TLR2/TLR4 expressed by trophoblast. Dysbiosis of placental microbiome increases the growth of pathogenic bacteria disrupting symbiosis among microbiota, trophoblast, and immune cells in the uterus, leading to inflammatory cascades that have been recognized in the pathogenesis of PE. Tregs, T regulatory cells; TLR, toll-like receptor; DC, decidual cell; LPS, lipopolysaccharide; LPP, lipoproteins; MAC, macrophage.

The placental microbiome has been reported to exert metabolic and immune regulatory functions in normal pregnancy as reported previously (Aagaard et al., 2014; Belkaid and Hand, 2014; Mor et al., 2017; Pelzer et al., 2017). Any perturbation in the composition or local balance of placental microbiome can lead to an unhealthy dysbiotic state, that may be associated with impaired immunoresponse/metabolic function resulting in adverse pregnancy outcomes particularly PE (Schoenmakers et al., 2018). Amarasekara et al., reported communities of pathogenic commensals such as *Variovorax* sp., *Anoxybacillus* sp., *Prevotella* sp., *Bacillus cereus, Escherichia* sp., *Klebsiella pneumoniae, Porphyromonas* spp., *Listeria* sp., *Salmonella* sp., and *Dialister* sp. in PE (Amarasekara et al., 2015) are in contrast distinct from the non-pathogenic commensals present in the placentae of normal pregnancies (Aagaard et al., 2014). The former was associated with infection, which possibly triggers inflammatory cells (Figure 3) and an imbalance between pro-angiogenic and anti-angiogenic factors. This could result in a series of events, such as impaired trophoblast function leading to endothelial dysfunction and placental hypoxia/ischaemia, which elevates maternal blood pressure causing PE (Amarasekara et al., 2015). In the light of this premise, it seems that the enrichment and diversity of commensals that are present in normal and pre-eclamptic pregnancies differ. The cause of this variation is not clear. Previous studies have linked adverse pregnancy outcomes, particularly preterm deliveries, to gut microbial remodeling and dysbiosis that precedes resultant loss of protective and homeostatic modulation (Shiozaki et al., 2014; Yoneda et al., 2016). Likewise, microbial products including bacterial DNA, which do not necessarily reflect live bacteria, reported to be sufficient to initiate adverse outcomes in pregnancy (Pelzer et al., 2017). Nevertheless, further studies are required to understand the cause of the variation in diversity and enrichment of the placental microbiome between normotensive and pre-eclamptic pregnancies, as it is currently unclear. This may lead to clinical predictive success and early diagnosis of PE.

**Figure 4:**
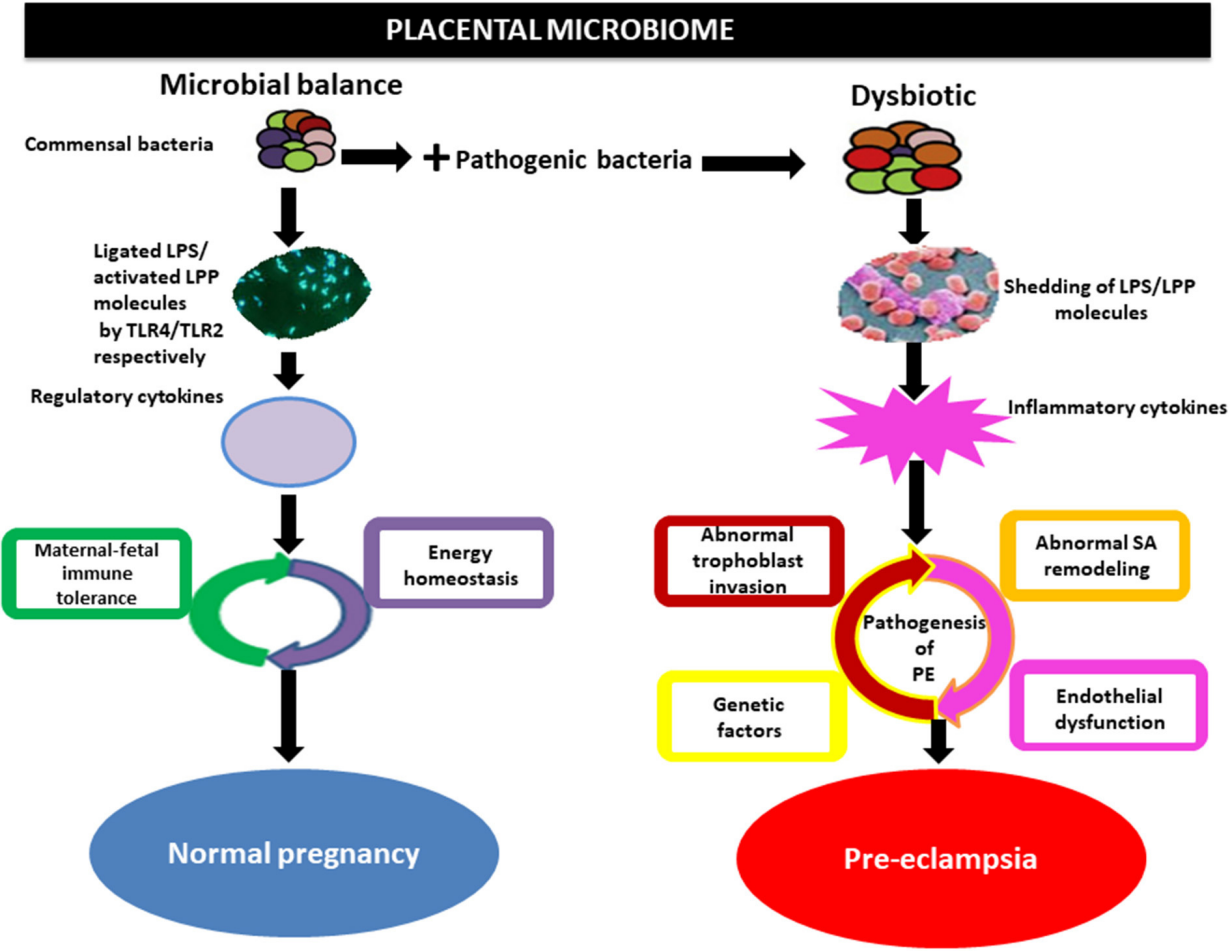
Implication of the placental microbiome in pre-eclampsia. Alteration of placental structures could result from the direct action of pathogenic bacteria through the release of lipopolysaccharide (LPS) or lipoproteins (LPP), which activates inflammatory cytokines via interactions with toll-like receptors (TLRs especially TLR4/TLR2). This invokes the pathogenic process of pre-eclampsia. Whereas, under physiological condition commensal bacteria release bacterial products such as LPS/LPP from gram-negative and positive bacteria, respectively, which is ligated by toll-like receptors (TLR4) and activated by TLR2 located on the surface of trophoblast. This in turns activates regulatory cytokines to promote tolerogenic microenvironment (maternal-fetal immune tolerance) and maintains energy homeostasis in normal pregnancy. SA, spiral artery.

In addition, the microbial products such as lipopolysaccharide (inflammagenic molecules) from the commensal bacteria including placental commensals have been found to play a major role in PE naturally or *in vivo* (Lin et al., 2012; Cotechini et al., 2014; Kell and Kenny, 2016). Lipopolysaccharide from gram-negative bacteria possibly activates TLRs particularly TLR3, TLR4, TLR7, and TLR8, inducing NF-kB (inflammatory mediator), while lipoproteins (LPP) or peptidoglycans from gram-positive bacteria activates TLR2, which leads through a cascade of intermediary steps to NF-kB activation (Figure 3) and thus, collectively initiating the pathogenesis of PE, including abnormal placentation and the maternal syndrome (Cotechini et al., 2014; Kell and Kenny, 2016). Although reports indicate that only humans are afflicted by PE (McCarthy et al., 2011; Xue et al., 2015), evidence from experimental animals, using a low dose infusion of lipopolysaccharide, show a pre-eclamptic-like syndrome, which includes hypertension, proteinuria, thrombocytopenia, increased anti-angiogenic factors, endothelial dysfunction, and elevated liver enzymes among others (Cotechini et al., 2014; Lip et al., 2017; Li et al., 2019). This implies that the lipopolysaccharide molecule is among the main mediators of PE (Figure 4). Moreover, placental microbial dysbiosis may also disrupt the metabolism of tryptophan and fatty acids, causing impaired maternal-fetal energy homeostasis, which possibly initiate or exaggerate pre-eclamptic events, especially during late gestation, hence, causing severe PE.

## Conclusion

An extensive review of the literature on the placental microbiome has been explored, including studies that have linked pregnancy complications especially PE to microbial alterations in the placenta of the host. The present review demonstrates that placental microbes play an important etiological role in PE by possibly shedding inflammagenic molecules such as lipopolysaccharide or lipoproteins, with a resultant inflammatory cascade that accompanies abnormal placentation and maternal endothelial cell activation.

## Future Research

Further studies with DNA-based techniques and well-controlled procedures are required to identify specific commensal bacteria that are possibly responsible for PE. Besides, the assessment of the clinical utility of early or pre-emptive antibiotics to reduce PE needs to be explored. We also like to propose a study that will determine the role of placental microbiome in early- and late-onset PE. It is also essential to combine the microbiome research with whole-genome sequencing, as well as metagenomics and metabolomics in order to adequately understand and interpret data regarding the potential effects of the microbiome on maternal immune response, metabolism, and epigenetics.

## Author Contributions

KO and IM conceived the project. KO drafted the review article under the supervision of IM and JM. KO, IM, JM, and YM read, revised, and approved the final review article. All authors contributed to the article and approved the submitted version.

## Conflict of Interest

The authors declare that the research was conducted in the absence of any commercial or financial relationships that could be construed as a potential conflict of interest.
